# A multi-compartment model for pathological connectomes

**DOI:** 10.1162/NETN.a.30

**Published:** 2025-10-30

**Authors:** Sara Bosticardo, Matteo Battocchio, Simona Schiavi, Andrew Zalesky, Cristina Granziera, Alessandro Daducci

**Affiliations:** Diffusion Imaging and Connectivity Estimation (DICE) Lab, Department of Computer Science, University of Verona, Verona, Italy; Translational Imaging in Neurology (ThINK), Department of Biomedical Engineering, University Hospital Basel, Basel, Switzerland; Melbourne Neuropsychiatry Centre and Department of Biomedical Engineering, University of Melbourne, Parkville, Victoria, Australia; Research Center for Clinical Neuroimmunology and Neuroscience Basel (RC2NB), University Hospital Basel, Basel, Switzerland; Department of Neurology, MS Center, University Hospital Basel, Basel, Switzerland

**Keywords:** Connectomics, Brain networks, Neurodegenerative diseases, Focal lesions, Multi-compartment models, Convex Optimization Modeling for Microstructure Informed Tractography

## Abstract

Brain connectivity analysis is pivotal to understanding mechanisms underpinning neurological diseases. However, current methodologies for quantitatively mapping the connectivity in vivo face challenges when focal lesions are present and can introduce strong biases in the estimates. We present a novel approach to address these challenges by introducing a multi-compartment description of the connectome, which explicitly incorporates lesion information during the estimation process. We extended the Convex Optimization Modeling for Microstructure Informed Tractography (COMMIT) framework to integrate an additional tissue compartment in voxels affected by pathology, allowing us to infer accurately the contributions of streamlines passing through lesions and to provide unbiased connectivity estimates. We evaluated the effectiveness of our approach on data from healthy subjects of the Human Connectome Project, in which we artificially introduced focal lesions to simulate pathology with varying levels of axonal damage. We also tested the performances obtained when comparing healthy subjects with patients affected by multiple sclerosis. Results demonstrate that our approach significantly enhances sensitivity to pathological changes even at low degeneracy levels compared with state-of-the-art techniques, thus representing a significant step forward to advance our understanding of neurodegenerative diseases.

## Author Summary

We present a novel microstructure-informed tractography method for estimating structural connectivity in the presence of focal pathologies, such as multiple sclerosis (MS). The model introduces a lesion compartment to accurately model the intra-axonal signal decay and refine streamline weights passing through lesions. The method was first evaluated using realistic simulations of axonal damage (44 subjects from the Human Connectome Project with simulated white matter lesions) and then tested on a dataset consisting of 84 healthy controls and 107 MS patients divided by disease phenotype. The results demonstrate that the proposed method effectively captures the pathology’s impact on structural connectivity, revealing significant differences in network metrics between healthy subjects and MS patients across both datasets.

## INTRODUCTION

Tractography allows mapping the structural connectivity of the brain in vivo and modeling it as a network, called *connectome*, where the nodes correspond to the gray matter regions and the edges are the reconstructed streamlines between them and represent the axonal connections in the white matter (WM) (Sporns, 2016; Sporns, Tononi, & Kötter, 2005; Yeh, Jones, Liang, Descoteaux, & Connelly, 2021; F. Zhang et al., 2022). The weights of the edges quantify the connection strength between the corresponding regions and should reflect the biological properties of the connections themselves, such as axonal density, cross-sectional area, axonal caliber, myelination, and so forth (Smith, Raffelt, Tournier, & Connelly, 2022; Sotiropoulos & Zalesky, 2019; F. Zhang et al., 2022).

**Figure 1.**
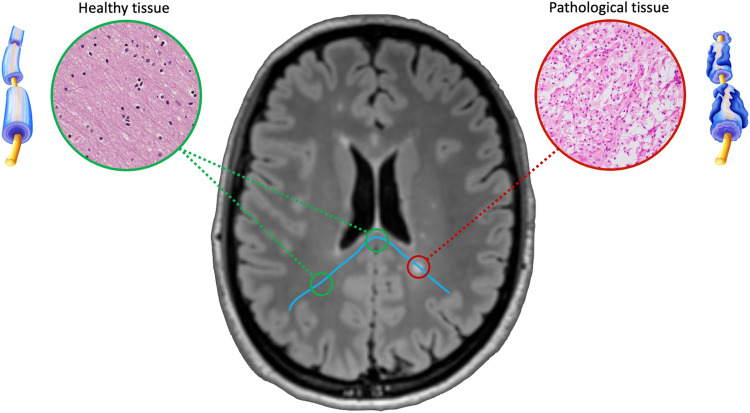
Example of local alterations of the neuronal tissue due to pathology: Normal-appearing WM is highlighted in green, whereas pathological tissue is in red. Clearly, if some fibers traverse both areas, as depicted here with the blue streamline, the fundamental assumption made by existing techniques, “a streamline represents a group of axons with the same trajectory and constant microstructural properties” cannot be satisfied in this situation; hence, assuming invariance of the microstructural parameters along a particular streamline will cause a mismatch between the streamline itself and the measured dMRI signal.

The simplest (and most adopted) approach for quantifying the strength of connections between two regions is to count the *number of streamlines* reconstructed between them. However, this does not provide an ideal biological marker of connectivity strength because tractography algorithms only follow the orientation of water molecules estimated by diffusion-weighted magnetic resonance imaging (dMRI) without making assumptions about the underlying axonal density or other biological features of interest (Jones, Knösche, & Turner, 2013; Smith et al., 2022; Sotiropoulos & Zalesky, 2019). Over the past decade, this limitation has stimulated a fast-growing interest in the community in gaining access to more biologically relevant properties of the connections, and several solutions have been developed, for example, Linear Fascicle Evaluation (Pestilli, Yeatman, Rokem, Kay, & Wandell, 2014), Convex Optimization Modeling for Microstructure Informed Tractography (COMMIT/COMMIT2) (Daducci, Dal Palù, Lemkaddem, & Thiran, 2015; Ocampo-Pineda et al., 2021; Schiavi, Ocampo-Pineda, et al., 2020), and spherical-deconvolution informed filtering of tractograms (SIFT/SIFT2) (Smith, Tournier, Calamante, & Connelly, 2013, 2015), which are commonly referred to as *microstructure informed tractography* (Daducci, Dal Palú, Descoteaux, & Thiran, 2016). Despite implementation differences, these methods attempt to quantify the strength of the connections by fitting the reconstructed tractogram, that is, set of streamlines, to the dMRI data and estimating a specific contribution, or weight, for each streamline that represents the effective intra-axonal cross-sectional area of the biological fibers along the corresponding trajectory. It should be noted that the estimation process behind all these methods is based on the hypothesis that such *contributions remain constant* along the streamlines; however, this *assumption is challenged* in the presence of diseases that locally alter the neuronal tissue (Figure 1).

Recent papers have highlighted some *critical consequences* of applying these methods to brains affected by focal pathology (Sarwar, Ramamohanarao, & Zalesky, 2019; Smith, Calamante, & Connelly, 2020a, 2020b; Zalesky, Sarwar, & Kotagiri, 2020a; Zalesky, Sarwar, & Ramamohanarao, 2020b). To start with, the diffusion signal from the intra-axonal compartment, reflecting the highly anisotropic and restricted diffusion of water molecules within axons, is influenced proportionally by both the volume of the lesion intersected and the degree of signal decay resulting from tissue degradation along a streamline passing through a pathological area (Zalesky et al., 2020b). Hence, the weight assigned by these algorithms to those streamlines passing through lesions should accurately reflect the actual signal decrease in the affected area, in analogy with the adage, “a chain is only as strong as its weakest link,” discussed in Zalesky et al. (2020a, 2020b). However, these algorithms do not assign weights to the affected streamlines that are proportional to the actual damage, because they attempt to match the local signal in all traversed voxels to avoid high fitting errors and lead to the overestimation of their connectivity strength. To further complicate matters, it is worth recalling that these methods rely on global error minimization, and their goal is to minimize the fitting error across all imaging voxel. Thus, they attempt *to compensate for this local mismatch* in the signal inside lesions by altering the streamline contributions, regardless of whether they are affected by pathology. As a result, even a localized error within a lesion can trigger compensatory mechanisms that may potentially bias the estimation of the entire connectome, including those bundles not affected by the pathology. These kinds of situations are commonly referred to as the “butterfly effect,” a metaphor that emphasizes the concept that in a complex system, even minor and seemingly insignificant events can have larger consequences elsewhere, similar to how the flap of a butterfly’s wings may eventually trigger a tornado far away.

**Figure 2.**
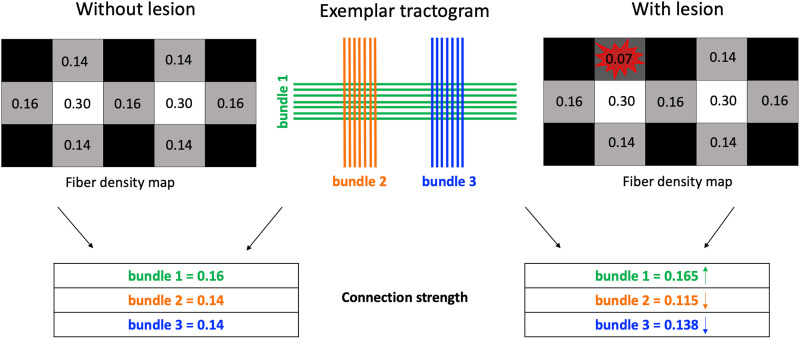
Synthetic example illustrating the *error-compensation mechanism* when using a generic microstructure-informed tractography method without accounting for lesions in the model. On the left, the connection strengths of the three exemplar bundles (middle panel) are reported, along with the corresponding fiber-density voxelwise map, in the absence of pathology, whereas on the right, we show these quantities as estimated when a focal lesion is present (red star-like shape). Due to the lack of considering the pathology in the model, the effect of the lesion is reflected not only on the bundles actually affected by pathology (underestimation, orange) but, potentially, also on the healthy ones (overestimation, green; underestimation, blue).

Figure 2 visually illustrates this *error-compensation mechanism* that can be triggered by a single pathological voxel if lesions are not considered in the model. The figure depicts a numerical simulation featuring two vertical bundles crossing a horizontal one at two distinct points. In the right panel, the upper part of Bundle 2 is affected by a lesion, while in the left panel, it is shown in a “healthy” state. Due to the signal loss in the pathological voxel (0.14 → 0.07), the estimated connection strength for Bundle 2 is 0.115, which is indeed lower compared with the same bundle when no pathology is present, that is, 0.14, even though this reduction in connection strength (0.14 → 0.115 ≈ −18%) does not accurately reflect the actual signal loss measured in the lesion (0.14 → 0.07 = −50%). Since Bundle 2 crosses Bundle 1, their total contribution in the crossing voxel should result in a measured signal equal to 0.30; however, as the estimated contribution of Bundle 2 is now 0.115, instead of 0.14, the contribution of Bundle 1 must be increased by ≈3% (0.16 → 0.165) to compensate for the underestimation of the signal in that voxel, that is, 0.115 + 0.16 < 0.30. However, Bundle 1 crosses Bundle 3 and so, in turn, the overestimation of Bundle 1 will have an impact also on Bundle 3: In fact, to minimize the mismatch between the signal reconstructed from tractography and the one measured in the voxel where they cross, that is, 0.165 + 0.14 > 0.30, its connection strength must be underestimated by ≈2% (0.14 → 0.138), even though Bundle 3 is not affected by pathology. It should be easy to see that this reasoning can be propagated at every crossing and that the misestimation of the connection strength can affect the whole connectome and can, thus, heavily limit its accuracy in modeling brain connectivity and its sensitivity to detect potential pathological conditions. We refer readers to the Supporting Information for a detailed walkthrough of the calculation in this example.

The *causes for this behavior* are twofold. First, current methods estimate the effective streamline contributions by fitting the tractogram to the measured data using classical multi-compartment signal models and assuming up to three compartments, that is, intra-axonal, extra-axonal, and isotropic, each representing specific patterns of water molecule movements in the brain tissue (Panagiotaki et al., 2012). However, an explicit compartment that accounts for the pathological tissue has never been considered so far. Second, only the streamline contributions corresponding to the intra-axonal compartment are considered when calculating the connectome, neglecting potential information from other tissues that may provide further insights into alterations of the connectivity due to pathology.

In this study, we propose a *multi-compartment model for pathological connectomes*, which introduces an explicit compartment for the estimation of the connectome to specifically handle the presence of pathological lesions. This requires two important modifications to the standard approach currently adopted: (a) augment the signal forward model to adequately fit the measured signal in all voxels, that is, both healthy and affected by pathology, and (b) compute the connectome by exploiting the information from all tissue compartments. This allows us to disentangle the contributions of the fibers from the actual tissue damage in every voxel and, in turn, to exploit this information for adjusting the cross-sectional area of the fibers when they pass through lesions, proportionally to the corresponding damage. We evaluated the effectiveness of our formulation in simulated data as well as in a population of patients affected by multiple sclerosis (MS). Our results demonstrate that our solution avoids introducing biases in the estimated streamline contributions and, consequently, in the connectome, thus providing more accurate and unbiased connectivity estimates.

## METHODS

### Microstructure Informed Tractography

In this paper, we focus on the COMMIT formulation (Daducci et al., 2015), but the proposed model is rather general, and it can be extended to other microstructure-informed tractography techniques (e.g., Pestilli et al., 2014; Schiavi, Ocampo-Pineda, et al., 2020; Smith et al., 2015). In a nutshell, COMMIT attempts to estimate the effective contribution, or weight, of each streamline in a tractogram by fitting them to the measured diffusion MR signal via a *global optimization problem*. The data in each image voxel are modeled as a linear combination of the signal originated by the different water pools that can be present in the tissue, whose actual contributions are recovered from the resulting linear system such that, at every brain location, they explain the measured signal at best:

$$\arg\;\min_{x\geq 0}{\left\|Ax-y\right\|}_{2}^{2},$$ (1)

where the vector **y** contains the measured dMRI data; the block matrix **A** = [**A**^*IA*^ | **A**^*EA*^ | **A**^*ISO*^] models the possible intra-axonal (IA), extra-axonal (EA), and free water (ISO) compartments within the tissue; and the coefficients **x** = [**x**^*IA*^ | **x**^*EA*^ | **x**^*ISO*^] to be estimated represent the corresponding effective contributions of each of them; see Daducci et al. (2015) for further details.

### Connectome Estimation

For each streamline *s*_*i*_ of the input tractogram, the corresponding coefficient $x_{i}^{IA}$ estimated by COMMIT represents the *effective intra-axonal cross-sectional area c_i_ of the axons* virtually described by that streamline, that is, *c*_*i*_ = $x_{i}^{IA}$. Hence, these values can be used to provide a quantitative and biologically plausible assessment of the brain’s structural connectivity (F. Zhang et al., 2022). In particular, the *connection strength* *C*_*b*_ of a bundle of interest *b*, which corresponds to a given edge in the connectome, is defined as the sum of the intra-axonal cross-sectional area of all streamlines belonging to *b*:

$$C_{b}=\sum_{i\in I_{b}}c_{i},$$ (2)

where the set *I*_*b*_ = {*i*_1_, …, *i*_*N*_*b*__} contains the indices of the streamlines belonging to bundle *b*, and *N*_*b*_ is the number of streamlines in *b*. The quantity *C*_*b*_ is considered a proportional estimate of axon count and, thus, represents a reasonable proxy for the information-carrying capacity of the biological pathway (F. Zhang et al., 2022). For this reason, it is called the fiber bundle capacity (Smith et al., 2022).

It is worth noting that although the matrix **A** in Equation 1 can implement multiple tissue compartments, that is, **A**^*IA*^, **A**^*EA*^, and **A**^*ISO*^, to date, the connectome is constructed using only the contributions **x**^*IA*^ of the streamlines, whereas the contributions from all other compartments, that is, **A**^*EA*^ and **A**^*ISO*^, are ignored and do not contribute to define the weights of the edges in the connectome. Thus, for the sake of simplicity, we will consider here a simplified setting where only intra-axonal contributions are considered in the signal forward model, as done in Schiavi, Ocampo-Pineda, et al. (2020), and their total amount in each voxel should match the local density of fibers, as estimated from dMRI (Kaden, Kelm, Carson, Does, & Alexander, 2016; H. Zhang, Schneider, Wheeler-Kingshott, & Alexander, 2012) or other modalities (Schiavi et al., 2022).

### Augmented Signal Forward Model to Account for Lesions

**Figure 3.**
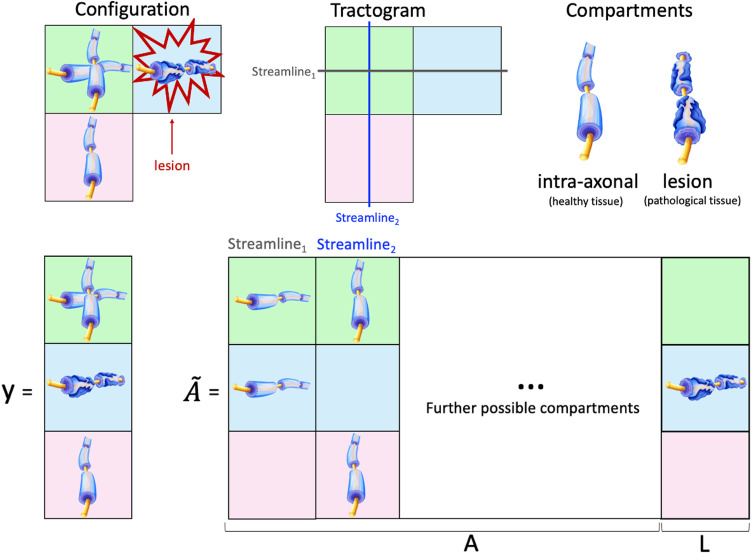
Cartoon example to illustrate how to augment the signal forward model of COMMIT to account for lesions. The upper panels display a simulated configuration consisting of two crossing bundles and a focal lesion, a possible tractogram reconstructed with tractography, and a two-compartment model to describe streamline contributions and lesion damage, respectively. Below are reported: the vector **y** with the data measured in the three voxels; the matrix $\tilde{A}$ encoding the streamline contributions, that is, **A**; and tissue damage in each affected voxel (only one in this case), that is, **L**.

As illustrated in Figure 3, we have enhanced the signal forward model of COMMIT with an *extra tissue compartment* to specifically capture potential axonal damage within the affected voxels. To achieve this, the matrix **A** has been augmented as $\tilde{A}$ = [**A** | **L**] and the submatrix **L** has one column for each pathological voxel (identified by a provided binary lesion mask), in which all entries are 0 except for the row corresponding to the pathological voxel that contains a negative contribution, that is, −1, to control the effective signal loss in that voxel caused by pathology. The *effective streamline contributions*, that is, **x**, and the *effective axonal damage* inside each pathological voxel, that is, **x**^**L**^, can then be estimated by solving the following augmented linear system:

$$\tilde{x}=\arg\;\min_{\tilde{x}\geq 0}{\left\|\tilde{A}\tilde{x}-y\right\|}_{2}^{2},$$ (3)

where $\tilde{x}$ = [**x** | **x**^**L**^]^*T*^ and the nonnegative coefficients **x**^**L**^ control the actual signal loss inside the lesions, as the matrix **L** contains negative contributions at the corresponding locations. This augmented signal model allows fitting the data accurately in all voxels and *prevents the compensatory mechanism* that may be triggered to reconcile any discrepancies inside lesions between the reconstructed tractogram and the actual signal measured.

### Connectome Estimation Accounting for Lesions

By decoupling the fiber contributions from the actual tissue damage into different compartments, we now have the possibility to gain insight into the real impact of focal lesions on the overall connectivity. However, as highlighted earlier, nowadays, the connectome is computed using only the intra-axonal contributions of the streamlines, and thus, this supplementary information on the tissue damage inside lesions would not be considered. For this reason, we argue that the *standard procedure for computing the connectome is inadequate* and must be improved to take advantage of both streamlines and lesions information.

First, we need to compute the *relative signal reduction inside each voxel v* due to the local axonal damage, as follows:

$$R\left(v\right)=\frac{\sum_{i\in I_{v}}x_{i}\;l\left(i,v\right)-x_{k\left(v\right)}^{L}}{\sum_{i\in I_{v}}x_{i}\;l\left(i,v\right)},$$ (4)

where **x** and **x**^**L**^ are the streamline contributions and voxelwise absolute signal losses, respectively, estimated with Equation 3; the set *I*_*v*_ contains the indices of the streamlines passing through voxel *v*; *l*(*i*, *v*) quantifies the length of streamline *s*_*i*_ inside *v*; and *k*(*v*) is the index in **x**^**L**^ corresponding to *v*. The quantity *R*(*v*) is always in [0, 1] ⊂ ℝ, with *R*(*v*) = 1 if the voxel is not affected by pathology and *R*(*v*) = 0 in case of complete axonal damage.

Second, we need to *relax the assumption of constant microstructural properties along streamlines* by allowing the cross-sectional area to vary along their course. To this aim, for each streamline *s*_*i*_ of the input tractogram, we iterate along its trajectory and modulate its local cross-sectional area according to Equation 4 when it passes through voxels affected by pathology, as follows:

$$c_{i}^{\prime }\left(t\right)=c_{i}\;R\left(s_{i}\left(t\right)\right),$$ (5)

where the parameter 0 ≤ *t* ≤ 1 defines the position along the streamline, $c_{i}^{\prime }$(*t*) is the locally modulated cross-sectional area at position *t*, and *s*_*i*_(*t*) = [*x*(*t*), *y*(*t*), *z*(*t*)] is the coordinate of the corresponding voxel. Then, inspired by the chain analogy suggesting that the connection strength should be “as strong as its weakest link” (Zalesky et al., 2020b), we define the *adjusted cross-sectional area of the streamline*, $\hat{c}$_*i*_, as the minimum value along its path of the locally modulated cross-sectional area, that is,

$${\hat{c}}_{i}=\min_{0\leq t\leq 1}c_{i}^{\prime }\left(t\right).$$ (6)

The quantity $\hat{c}$_*i*_ represents the weakest link of the axonal connection described by streamline *s*_*i*_ and is then used in place of *c*_*i*_ to compute the connection strength of the corresponding bundle with Equation 2 as usual.

By introducing the possibility to refine the cross-sectional area of the streamlines proportionally to the axonal damage estimated from the newly introduced compartment, that is, Equation 6, the connectome is no longer solely determined by the intra-axonal contribution of the streamlines, as currently done by existing techniques, but now, it incorporates additional information about the local tissue damage inside lesions. For this reason, we will refer to this new approach of computing the connectome with the term *multi-compartment connectome*.

### Evaluation in Realistic Numerical Simulations of Tissue Damage

**Figure 4.**
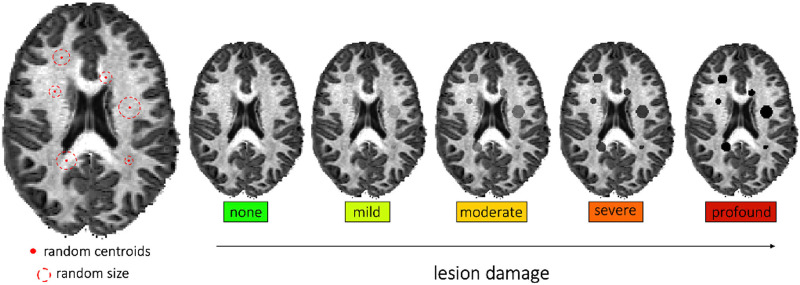
Axial slices of T1 images showing lesions modeled as spherical objects within the WM in which the signal is locally altered by the pathology. The positions and extents of the lesions were randomly set such that the total amount of “pathological” voxels covers 5% of the WM volume as reported in the literature for samples of MS patients. We simulated five levels of tissue damage: none (i.e., healthy subject), mild (25% reduction), moderate (50% reduction), severe (75% reduction), and profound (100% reduction).

We quantitatively assessed the *sensitivity of multi-compartment connectomes to detect anomalies in the connectivity* due to pathology using realistic simulations of tissue degeneration in 44 subjects from the Human Connectome Project (HCP) test–retest dataset acquired using a 3T system (Van Essen et al., 2013). We chose to use this dataset because it allows us to compare the connectivity of healthy subjects with the connectivity of a pathological model for the same subject. By modeling the pathology in each subject, we could systematically assess the model’s sensitivity at different levels of axonal damage. As illustrated in Figure 4, for each subject, we created a binary mask to define the position and extent of virtual lesions, which were modeled as spheres and whose centroids were randomly placed within the WM and then randomly dilated until they covered 5% of the WM, as typically observed, for example, in patients affected by MS (Ouellette et al., 2020). To simulate tissue damage, we scaled the signal in each voxel inside this mask according to the *five levels of degeneration*: “none” (0% reduction, i.e., healthy subject), “mild” (25% reduction), “moderate” (50% reduction), “severe” (75% reduction), and “profound” (100% reduction, i.e., tissue completely corrupted).

For each degree of tissue degeneration, we computed *standard and multi-compartment connectomes*. To define the nodes, we segmented cortical and subcortical tissues into 85 regions according to the standard Desikan–Killiany atlas (Desikan et al., 2006) using FreeSurfer 6.0 (Fischl, 2012). To define the edges, we reconstructed 3 million streamlines using the iFOD2 algorithm with anatomical priors (Smith, Tournier, Calamante, & Connelly, 2012), seeding from the gray matter-white matter interface and propagating the streamlines with the backtrack option. Finally, the intra-axonal signal fraction map used in Equations 1 and 3 to quantify the edge weights, that is, vector **y**, was estimated using the Spherical Mean Technique (Kaden et al., 2016; Kaden, Kruggel, & Alexander, 2016).

To evaluate the sensitivity of multi-compartment connectomes to *detect anomalies in the connectivity* due to pathology, we conducted a cross-sectional analysis using a permutation-based statistical test implemented in Scipy (Virtanen et al., 2020) to compare the two approaches for the estimation of connectomes, that is, “without” and “with” lesion modeling. We focused on three network measures typically used in clinical applications (Bosticardo et al., 2022; Charalambous et al., 2019; Fleischer et al., 2019; Griffa, Baumann, Thiran, & Hagmann, 2013; Kocevar et al., 2016; Llufriu et al., 2017; Pagani et al., 2020; Schiavi, Petracca, et al., 2020): mean strength, global efficiency, and modularity (Rubinov & Sporns, 2010), calculated using the Brain Connectivity Toolbox for Python (https://github.com/aestrivex/bctpy). For each level of axonal damage, we performed 5,000 permutations by randomly dividing the 44 subjects into healthy controls (HC) and patients for each permutation, and the reported *p* values correspond to the proportion of tests where the computed *p* value exceeded 0.05.

### Evaluation in Patients Affected by MS

We also assessed the effectiveness of the multi-compartment connectomes in a cohort of 84 HC (age 37.5 ± 13.0 years, 45 females), 70 relapsing-remitting MS patients (RRMS) (age 37.8 ± 11.1 years, 44 females, 2 median Expanded Disability Status Scale) and 37 progressive MS patients (PMS) (age 59.8 ± 8.36 years, 17 females, 5 median Expanded Disability Status Scale). The inclusion criteria for the enrollment of MS patients included the following: age between 18 and 75 years, MS diagnosis fulfilling the McDonald criteria (Thompson et al., 2018), absence of neurological or psychiatric disease other than MS, and absence of contraindications in subjects for MRI. The ethical review committee of the University Hospital Basel (Institutional Review Board of Northwest Switzerland) approved the study, and all participants entered the study after written consent. All subjects underwent MRI on a 3T system (Prisma; Siemens Healthcare, Erlangen, Germany) with a 64-channel head and neck coil. The acquisition protocol included the following: 3D Fluid Attenuated Inversion Recovery (TR/TE/TI = 5,000/386/1,800 ms) and Magnetization Prepared 2 Rapid Acquisition Gradient Echoes (TR/TI1/TI2 = 5,000/700/2,500 ms), both with 1-mm isotropic spatial resolution; multishell multiband spin-echo diffusion (TR/TE/impulse duration/time between impulses = 4,500/75/19/36 ms); and 1.8-mm isotropic spatial resolution with *b* values of 700/1,000/2,000/3,000 s/mm^2^ and 6/20/45/66 diffusion directions per shell, respectively, in addition to 12 measurements at *b* value of 0 s/mm^2^ with both anterior to posterior and reversed phase encoding.

MS lesions detectable in FLAIR images were semiautomatically segmented with an automatic in-house deep learning-based method (La Rosa et al., 2020), followed by manual correction. Then, lesion masks were filled on the MP2RAGE images to improve the registration and segmentation steps on the patients’ images (Griffiths-King et al., 2023). To ensure accurate spatial alignment, we employed the boundary-based linear registration tool implemented in FSL (Jenkinson, Bannister, Brady, & Smith, 2002) to register all segmented masks to the diffusion space. dMRI data were preprocessed to reduce artifacts from noise (Veraart, Fieremans, & Novikov, 2016; Veraart, Novikov, et al., 2016), eddy currents (Andersson & Sotiropoulos, 2016), motion and EPI distortions (Andersson, Skare, & Ashburner, 2003; S. M. Smith et al., 2004) using MRtrix3 (Tournier et al., 2019), and FSL (Jenkinson, Beckmann, Behrens, Woolrich, & Smith, 2012; Woolrich et al., 2009). dMRI images were corrected for B1 field inhomogeneity using the N4 algorithm implemented in ANTs (Tustison et al., 2010) and upsampled to match the 1-mm isotropic resolution of MP2RAGE. For consistency with the numerical simulations, we adopted the same pipeline described above for the definition of connectome nodes, edges, and edge weights, as well as for the computation of the network measures from the estimated connectomes.

The statistical analysis was conducted using R (R Core Team, 2013). To address potential outliers, we utilized linear robust models (Koller & Stahel, 2011). Differences in density between the two groups of subjects (HC and the entire MS patient sample) were tested with age, gender, and WM volume included as covariates in the model. Density was found to differ significantly between the two groups (*p* < 0.001). Consequently, in line with the findings reported in Schiavi, Petracca, et al. (2020) and van Wijk, Stam, and Daffertshofer (2010), we included this variable as a confounding factor in the model. Our null hypothesis posits that there are no differences in network metrics among controls and patients, subdivided into RRMS and PMS groups, when extracted from a connectome weighted using either the standard method or the multi-compartment connectome.

## RESULTS

### Evaluation in Realistic Numerical Simulations of Tissue Damage

**Figure 5.**
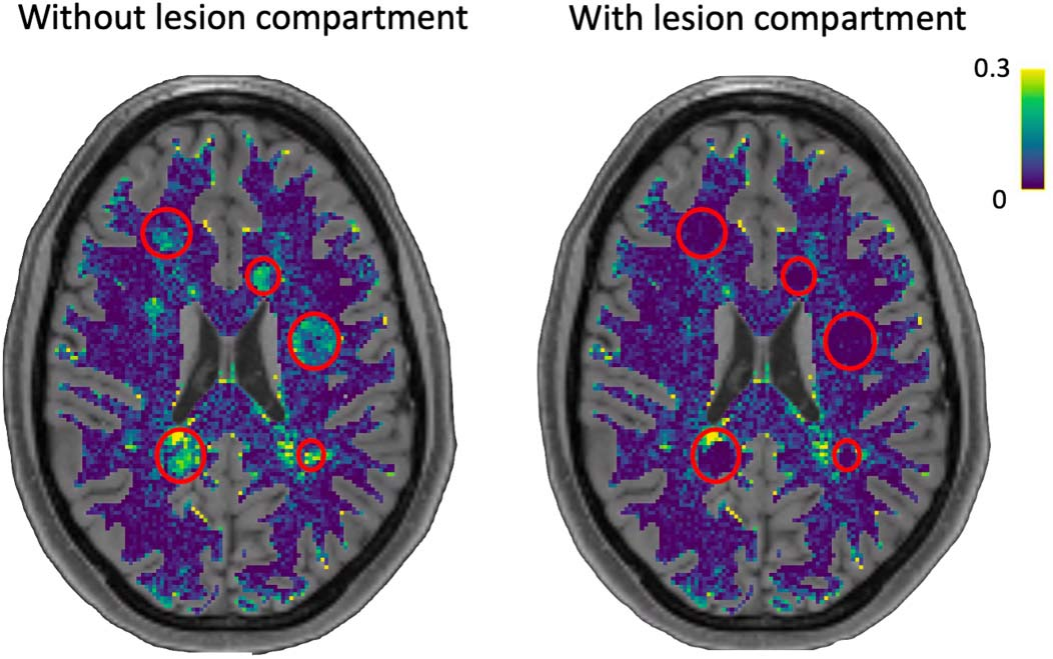
Root mean squared error of the fit without (left) and with (right) explicit modeling of the lesions. The red circles indicate the position and extent of the simulated lesions, clearly showing a modest error in the fit when using the model without considering an explicit compartment for the lesioned tissue, with potentially severe consequences on all estimated coefficients.

To demonstrate the effectiveness of the proposed framework, we compared the standard connectomes (i.e., without lesion modeling) with the multi-compartment connectomes (i.e., with lesion modeling). In Figure 5, we present the root mean square error of the signal fitted by the two models in a representative axial slice where the areas affected by the lesions are identified by the red circles. It can be observed that in voxels affected by the pathology, the error is modest when utilizing the standard connectomes without the lesion compartment to model the signal. However, with the inclusion of our extension, the error becomes comparable with that of other WM voxels.

**Figure 6.**
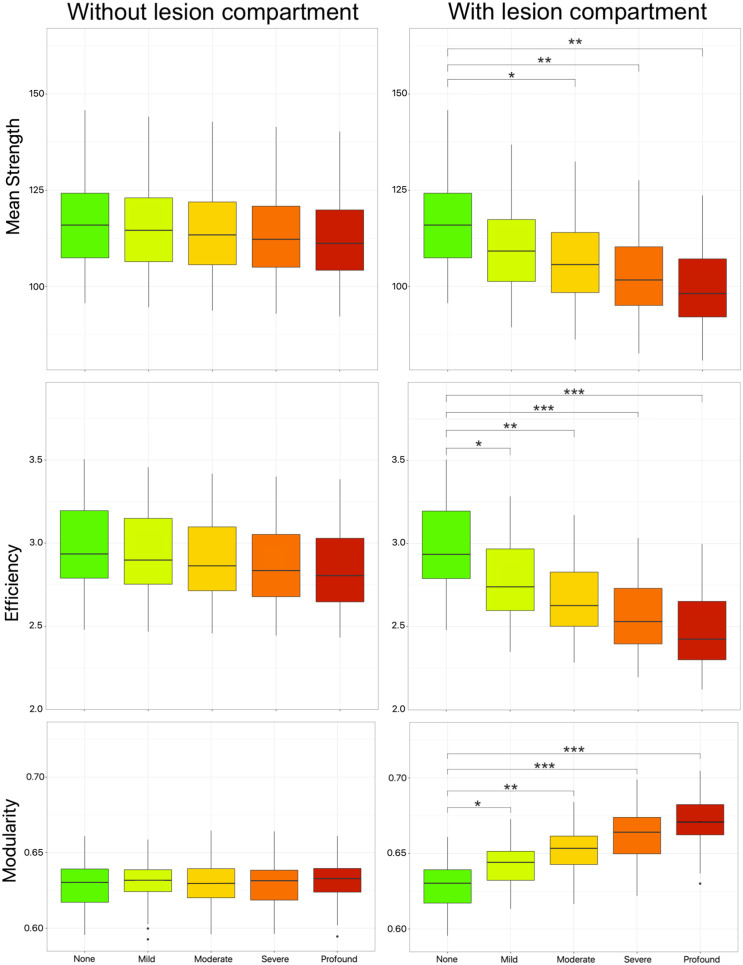
Comparison of global network measures as estimated by state-of-the-art connectomics approaches (left, without lesion compartment) and the proposed multi-compartment connectomes (right, with lesion compartment), as a function of the degree of lesion damage. The asterisks in the figure represent the level of significance (*p ≤ 0.05, **p ≤ 0.01, ***p ≤ 0.001). The results of the permutation test are reported in Table 1.

The box plots in Figure 6 depict three global network metrics extracted from the connectomes calculated using the two methodologies. Specifically, the left panel shows the network metrics derived from connectomes weighted with the standard model (i.e., without lesion modeling), while the right panel displays those obtained using the multi-compartment connectome (i.e., with lesion modeling). The plots represent HCP subjects modeled according to five levels of simulated axonal damage. Statistical significance, indicated by asterisks, was determined using the permutation test, with the results detailed in Table 1. Notably, the differences in network metrics among the groups are practically negligible when lesion modeling is not applied. In contrast, the differences between the five groups become more pronounced and evident when using the multi-compartment connectomes.

**Table 1.**
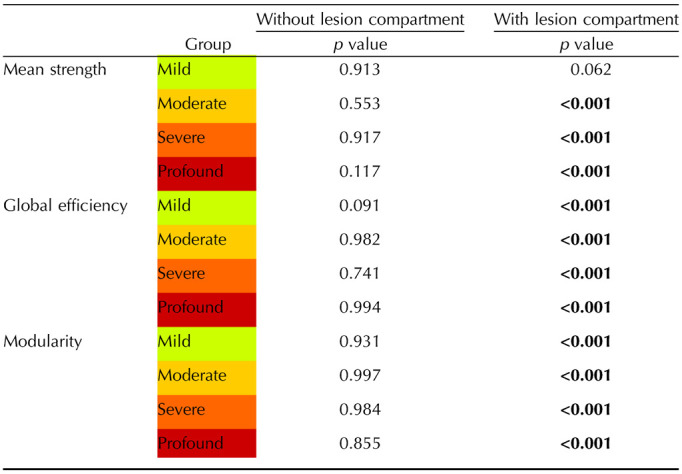
The table presents the results of the permutation test conducted to compare HC with patients modeled at different levels of axonal damage

The results shown in the table are visualized in the plots in Figure 6. We reported the *p* value computed as the proportion of *p* values above 0.05 out of the total number of iterations. Significant *p* values are reported in **bold**.

In Table 1, the results of the permutation test are presented, where subjects were randomly divided into different groups based on the degree of axonal damage. The results highlight what was apparent from the plots in Figure 6. Specifically, when not considering the lesion compartment, there are no statistically significant differences between groups with different levels of axonal damage and healthy subjects. However, when considering the lesion compartment, efficiency and modularity differ between HC and patients with simulated pathology at levels ranging from mild to profound, while mean strength shows differences between HC and patients with simulated pathology at levels from moderate to profound.

### Evaluation in Patients Affected by MS

**Figure 7.**
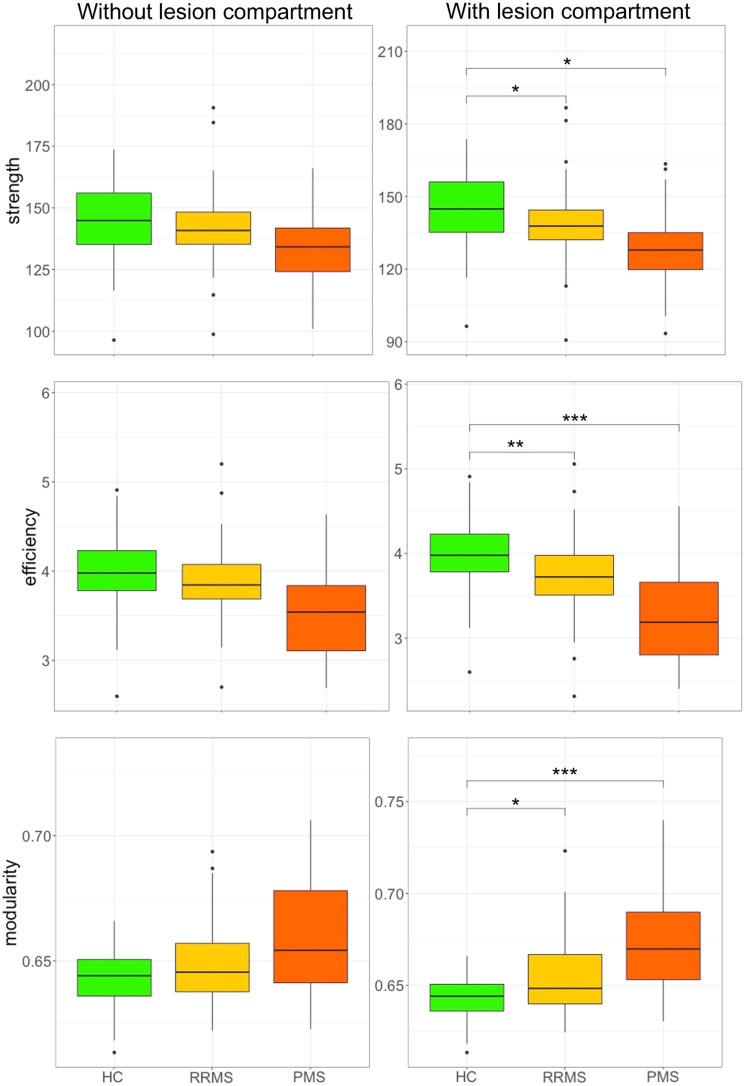
Comparison of global network measures of the connectomes estimated using the standard method (without lesion compartment) and our proposed approach (with lesion compartment). Asterisks represent the significance level (*p ≤ 0.05, **p ≤ 0.01, ***p ≤ 0.001) computed using a robust linear model corrected for sex, age, network density, and WM volume.

The box plots in Figure 7 illustrate the network metrics extracted from the connectomes calculated using the standard version of the model (i.e., without lesion compartment) and the multi-compartment connectome (i.e., with lesion compartment). Asterisks in the figure represent the level of significance, calculated using a robust linear model corrected for age, sex, network density, and WM volume, as described previously.

In this analysis, the connectomes estimated using the standard approach appear to lack sensitivity to pathology, while the multi-compartment connectomes are capable of identifying significant differences for all three tested network metrics between patients and controls. Specifically, significant differences were observed for mean strength (HC vs. RRMS, *p* = 0.05; HC vs. PMS, *p* = 0.03), efficiency (HC vs. RRMS, *p* = 0.007; HC vs. PMS, *p* < 0.001), and modularity (HC vs. RRMS, *p* = 0.03; HC vs. PMS, *p* < 0.001). The results of the analysis are reported in Table 2.

**Table 2.**
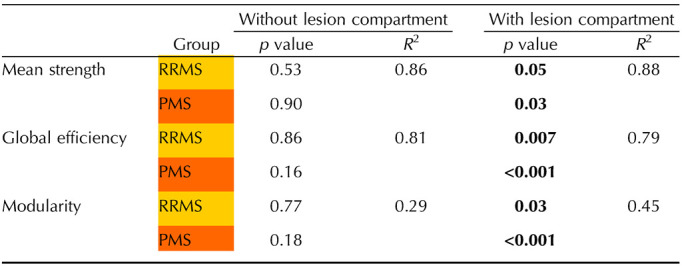
The table reports the group analysis results performed using the standard model (i.e., without lesion compartment) and the multi-compartment connectome (i.e., with lesion compartment)

The analyses were conducted using a robust linear model corrected for age, sex, density, and WM volume. Tests that reach statistical significance are highlighted in **bold**.

The experiments conducted on these data serve as additional validation of the model’s applicability. However, as this study aims to present a novel framework rather than an application study, corrections for multiple comparisons were not performed.

## DISCUSSION

In the last decade, innovative methods have been developed to achieve a better understanding of structural brain connectivity (Daducci et al., 2015; Ocampo-Pineda et al., 2021; Pestilli et al., 2014; Schiavi, Ocampo-Pineda, et al., 2020; Smith et al., 2013, 2015). This class of methods is called microstructure-informed tractography (Daducci et al., 2016), and it allows the integration of microstructural properties with tractography, enhancing the biological accuracy of the estimations. Despite the promising results obtained in different population studies conducted using these techniques, concerns have been raised about their applicability in cases of focal pathologies (Sarwar et al., 2019; Smith et al., 2020a, 2020b; Zalesky et al., 2020a, 2020b). Indeed, these methods rely on the assumption that a streamline represents a group of axons following a common trajectory with constant microstructural properties along their path. However, in the presence of focal pathology, this assumption is compromised, triggering compensation mechanisms in the estimation of the connectivity due to signal mismatches in voxels affected by pathology, introducing biases even for bundles unaffected by pathology.

In this work, we extended the microstructure-informed tractography framework COMMIT by introducing an additional compartment that accounts for signal loss in voxels affected by the pathology. This approach addresses the issues highlighted in Sarwar et al. (2019) and Zalesky et al. (2020a, 2020b). By changing the modeling of signal decay in specific voxels, our method aims to mitigate the compensation mechanisms and prevent the alteration of connection strength in bundles unaffected by pathology. Furthermore, the incorporation of information about the lesions’ contributions to the estimated signal permits the calculation of a relative signal reduction within each voxel. This allows us to appropriately scale the weights of streamlines traversing pathological voxels in proportion to the measured axonal damage. Consequently, the proposed approach enables the estimation of a connectome that accounts not only for intra-axonal contributions (as achieved with the standard method) but also for the impact of axonal damage, resulting in what we define as the *multi-compartment connectome*.

Our results demonstrate that introducing the lesion compartment improves sensitivity to pathology, both in realistic numerical simulations and in patients with MS. In the numerical simulations, the multi-compartment model detected significant differences between HC and subgroups with different levels of modeled axonal damage. In contrast, the standard method failed to identify changes in structural connectivity, even in cases of the most severe modeled axonal damage. To further validate our results, we repeated the analyses using different threshold levels of network density (≈50% and ≈25%), which are commonly referenced in the literature. The model demonstrated robustness, yielding similar outcomes to those observed in the original results, where the connectomes had a density of about 70%. Furthermore, we tested the method by modeling different lesion volumes while keeping the axonal damage within them fixed. Again, the method proved to be robust, confirming the initial experiments. The results of these different experiments are presented in the Supporting Information.

Results from the application on a real dataset of MS patients further support and confirm these findings. Using multi-compartment connectomes, we detected significant changes in global network metrics in RRMS and PMS patients compared with HC—changes that the standard method failed to capture. This limitation of the standard method arises from its inconsistency with the chain principle: It adjusts the overall strength of the chain to maintain uniform consistency between the chain’s strength and that of its individual links. Such an approach can obscure the effects of pathology, much like masking the presence of the weakest link in a chain.

In summary, our findings highlight that multi-compartment connectomes provide a more accurate and biologically relevant representation of brain connectivity in the presence of focal pathologies, such as MS. This approach offers valuable insights into how axonal damage affects the global brain network and may enhance our understanding of the disease’s effects on connectivity metrics. Furthermore, these results emphasize a critical point with significant clinical implications. When focal pathology is present, using microstructure-informed tractography methods without explicitly modeling the lesions can hide the true impact of the pathology on structural connectivity. This can result in misleading conclusions about the extent and nature of connectivity changes induced by the disease, ultimately compromising the reliability of connectivity-based biomarkers and their interpretability in clinical settings.

An advantage of the proposed approach is that it can be applied to any microstructure-informed tractography method described in the literature (Daducci et al., 2015; Ocampo-Pineda et al., 2021; Pestilli et al., 2014; Schiavi, Ocampo-Pineda, et al., 2020; Smith et al., 2013, 2015) by adjusting the signal modeling according to Equation 3 and rescaling the streamline weights according to Equation 6. In this work, we specifically focused on implementing it within the COMMIT framework due to its modular and flexible architecture. However, it is worth noting that our attempt to model damage arising from localized lesions is not the first approach proposed in the literature to improve the connectivity estimation performed through microstructure-informed tractography methods. Smith and colleagues also proposed modifying the original SIFT2 method (Smith et al., 2015) to avoid overestimating the connectivity of bundles affected by pathology (Smith, Calamante, Gajamange, Kolbe, & Connelly, 2020). More in detail, they introduced a variable *ω* calculated by multiplying the SIFT2 proportionality coefficient by the track density, all divided by the fiber density (Smith, Calamante, Gajamange, et al., 2020). This coefficient allows for the adjustment of connectivity in bundles affected by pathology based on the level of axonal damage, in a manner similar to how we employ the relative signal reduction within each voxel. The key difference between the methodology introduced by Smith and colleagues and our approach lies in how lesion-related information is incorporated. In our method, the lesion compartment is integrated directly into the model-fitting process, rather than being applied a posteriori during the computation of the resulting weighted connectomes. We argue that this integration grants to avoid the cascade of compensation mechanisms triggered to minimize errors in lesion-affected voxels, which can influence all bundles, regardless of whether they are affected by pathology. In contrast, relying *solely* on a weight-scaling factor, as in Smith’s approach, does not prevent the erroneous estimation of healthy bundles, such as those intersecting with the pathological ones. It is relevant to mention that both proposed methodologies focus solely on WM lesions, as gray matter lesions are less relevant to our study given that the analyzed tracts pass through WM.

This paper presented applications of the proposed method related to MS. However, our formulation is very flexible and can also be applied to tissue lesions induced by other diseases. In fact, the formulation of COMMIT can accommodate different types of input data other than dMRI, such as quantitative T2 and myelin-sensitive maps (Barakovic, Girard, et al., 2021; Barakovic, Tax, et al., 2021; Bosticardo et al., 2024; Leppert et al., 2023; Schiavi et al., 2022). Consequently, the method can be easily adapted to different pathological scenarios by selecting the most appropriate microstructural map for the fit of the model with respect to the pathology, broadens the potential applications of the model, making it suitable for a wide range of studies aimed at characterizing structural connectivity and its relationship with specific tissue parameters.

Moreover, in our formulation, a lesion is not considered a single entity; instead, every voxel within the binary mask of the lesion is considered independently inside the optimization process. For instance, if edema surrounding a lesion is to be considered, this should be explicitly included in the mask, but in the current implementation, this should be done manually by the user. Indeed, a key limitation of the proposed framework is its reliance on a lesion mask as input to accurately identify areas of pathology. This dependency may restrict its applicability in cases where lesion masks are unavailable or challenging to obtain. In future studies, we aim to address this limitation by refining the model to enable the automatic detection of voxels characterized by abnormal signal degradation, eliminating the need for a predefined WM lesion mask. Such advancements would not only enhance the model’s clinical applicability but also extend its use to a broader range of pathologies, including those where lesion segmentation is not a standard clinical practice. Beyond these technical improvements, we also aim to explore the clinical utility of multi-compartment connectomes more comprehensively. Future studies will investigate the relationship between network metrics and clinical scores, as well as examine additional phenotypic correlations. Indeed, while this work presents some preliminary results on a cohort of MS patients, future applications will focus on a more extensive exploration, incorporating a broader range of data to validate the framework further and assess its full potential.

## CONCLUSIONS

Microstructure informed tractography allows enriching the characterization of the human connectome with quantitative and biologically informative features of WM connections, such as axonal density or myelination. Existing methods are grounded on the hypothesis that these microstructural properties remain constant along the reconstructed streamlines. However, this assumption clearly breaks in pathologies that locally affect the neuronal tissue, such as MS, introducing biases in the estimated connectivity that can potentially conceal the effect of pathology. In this paper, we proposed to augment the classical description of the connectome with an extra compartment that explicitly accounts for tissue damage inside the lesions and allows for discontinuities of the microstructural environment along the streamlines. Results on synthetic and human brain data showed that multi-compartment connectomes deliver unbiased connectivity estimates in case of focal pathology and significantly enhance sensitivity to pathological changes compared with existing techniques, even at the early stages of the disease. Our work represents a significant step forward in the study of the impact of focal lesions on overall connectivity and, thus, advancing our understanding of neurodegenerative diseases.

## SUPPORTING INFORMATION

The processed data supporting the conclusions of this article will be made available by the authors. The code used to run the model presented in this study, along with an example script, is available on GitHub (https://github.com/daducci/COMMIT/wiki/Multicompartment-model-for-pathological-connectomes). Supporting information for this article is available at https://doi.org/10.1162/NETN.a.30.

## AUTHOR CONTRIBUTIONS

Sara Bosticardo: Conceptualization; Formal analysis; Investigation; Methodology; Software; Visualization; Writing – original draft; Writing – review & editing. Matteo Battocchio: Investigation; Methodology; Software; Writing – review & editing. Simona Schiavi: Formal analysis; Investigation; Supervision; Writing – review & editing. Andrew Zalesky: Formal analysis; Investigation; Writing – review & editing. Cristina Granziera: Conceptualization; Formal analysis; Funding acquisition; Resources; Supervision; Validation; Writing – review & editing. Alessandro Daducci: Conceptualization; Funding acquisition; Investigation; Methodology; Project administration; Resources; Software; Supervision; Writing – original draft; Writing – review & editing.

## COMPETING INTERESTS

S.S. is an employee of ASG Superconductors Genoa, but this research was conducted in the absence of any commercial or financial relationships that could be construed as a potential conflict of interest. C.G. is an employee of University Hospital Basel (USB) and the Research Center for Clinical Neuroimmunology and Neuroscience (RC2NB); her institutions have received fees from the following, which were used exclusively for research support: Siemens, GeNeuro, Genzyme-Sanofi, Biogen, and Roche. C.G.’s institutions have also received advisory board and consultancy fees from Actelion, Genzyme-Sanofi, Novartis, GeNeuro, Merck, Biogen and Roche; as well as speaker fees from Genzyme-Sanofi, Novartis, GeNeuro, Merck, Biogen, and Roche.

## FUNDING INFORMATION

This work was supported by the Swiss National Science Foundation (SNSF) grants PP00P3_206151 and 32003BE_232739, and by the PRIN 2022 research program framework, project “FAITH” (CUP B53D23018930006), PNRR M.4 C.2 I.1.1, MUR, Next Generation EU.

## Supplementary Material



## TECHNICAL TERMS

Connectome: Mathematical representation of brain connections, where edge weights reflect intra-axonal contributions from dMRI-based tractography.
Cross-sectional area: The effective intra-axonal area represented by a streamline, reflecting the size and density of axons within a fiber bundle.
Butterfly effect: A metaphor describing how small changes in a complex system can lead to significant, far-reaching consequences elsewhere.
Global optimization problem: Finding the best solution from all possible options in a complex system, avoiding suboptimal local solutions.
Fiber bundle capacity: The total contribution of a fiber bundle to the diffusion signal, serving as a proxy for its information-carrying capacity.
Multi-compartment connectome: Mathematical representation of brain connections, with edge weights including both intra-axonal and lesion compartment contributions.
