# Supplementary material for: A multi-compartment model for pathological connectomes
Source: Netw Neurosci. 2025 Oct 30;9(4):1245–63. doi: 10.1162/NETN.a.30 (PMC12594486; doi:10.1162/NETN.a.30)
Supplement: Supplementary file 1 [file netn-9-4-1245-s001.pdf]

SUPPORTING INFORMATION

Walk through the calculation of the example

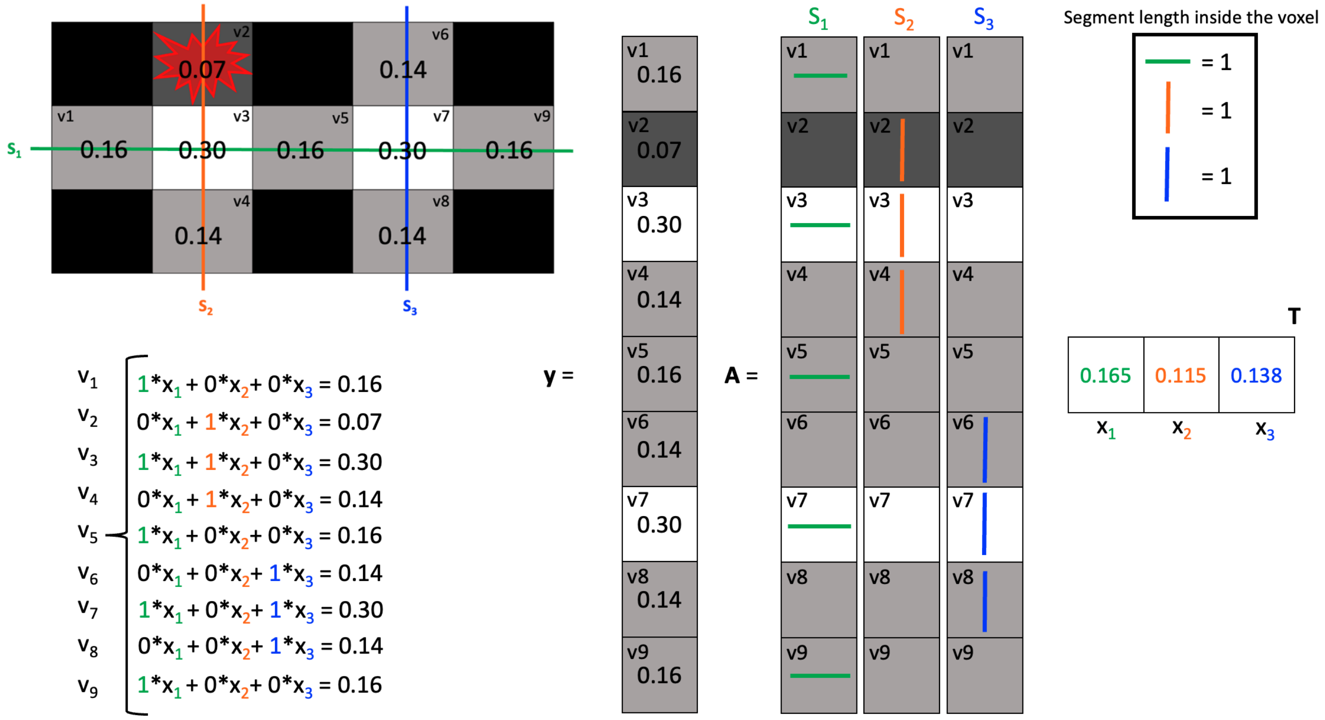

Explain negative contribution:

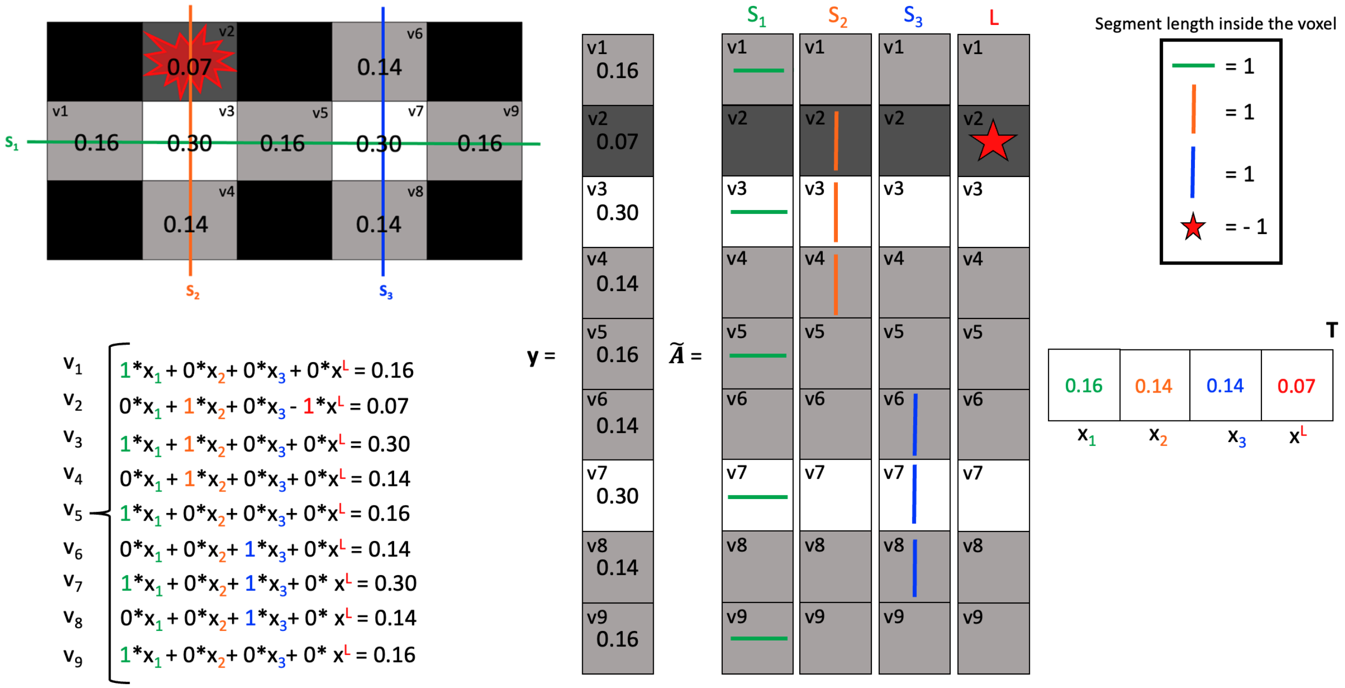

Explain R:

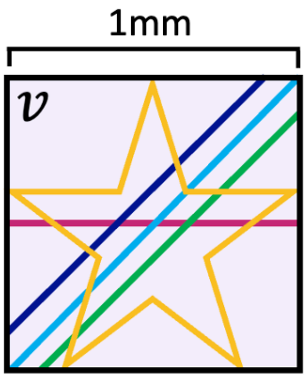

$$R(v) = \frac{(x_i l(i,v) + x_i l(i,v) + x_i l(i,v) + x_i(i,v)) - x_L(\star, v)}{(x_i l(i,v) + x_i l(i,v) + x_i l(i,v) + x_i(i,v))}$$

An illustrative example of the *relative signal reduction* computed in a voxel v according to Eq. **??**. Each x_i represents the effective cross-sectional area of streamline s_i estimated by COMMIT, and $l_(i, v)$ is the corresponding length inside voxel v.

Different Density thresholding:

We tried different network density thresholding to test the robustness of our method. We set the weakest connections in the healthy subjects to zero so that the network density remains at a percentage equal to 25% and 50%. The original density was 70%. Then, we recomputed the network metrics on the new thresholded connectomes.

Fifty percent thresholding

The plot in Figure 1 and the results in Table 1 correspond to the network metrics computed after setting the weakest connections of the HC subjects to zero, achieving a network density of approximately 50%.

		Without lesion compartment	With lesion compartment
	Group	p-value	p-value
MEAN STRENGTH	mild	0.975	0.234
	moderate	0.982	**0.025**
	severe	0.796	**<0.001**
	profound	0.581	**<0.001**
GLOBAL EFFICIENCY	mild	0.999	<0.443
	moderate	0.719	**<0.001**
	severe	0.769	**<0.001**
	profound	0.930	**<0.001**
MODULARITY	mild	0.997	**0.033**
	moderate	0.998	**<0.001**
	severe	0.417	**<0.001**
	profound	0.518	**<0.001**

Table 1. The table present the results of the permutation test conducted on the connectomes thresholded by the 50%. The results shown in the table are visualized in the plots in Figure 1.

Twenty-fifth percent thresholding

The plot in Figure 2 and the results in Table 2 correspond to the network metrics computed after setting the weakest connections of the HC subjects to zero, achieving a network density of approximately 25%.

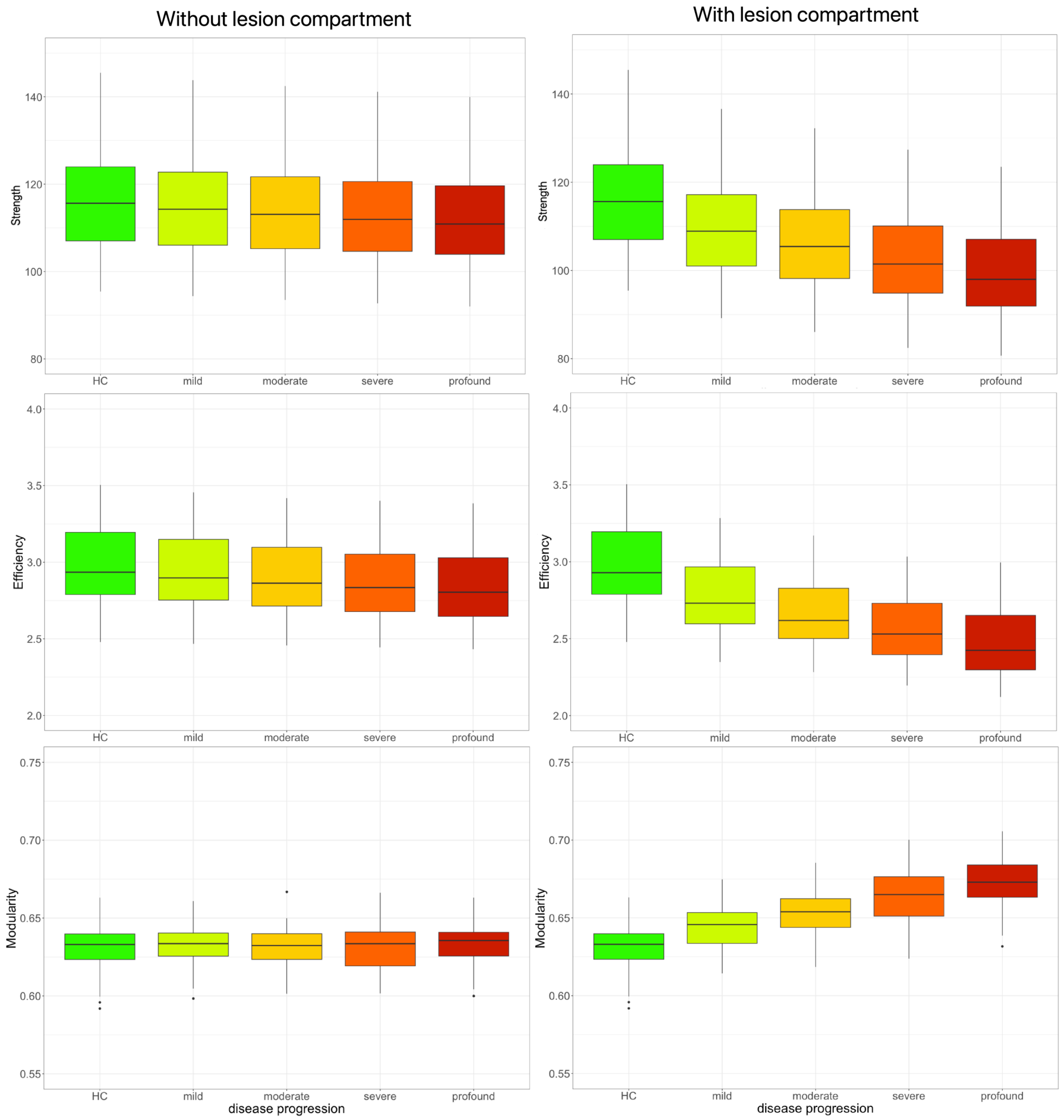

1 **Figure 1.** Comparison of global network metrics estimated with and without the lesion compartment on connectomes thresholded at 50%. The results of the
2 permutation test are presented in Table 1.

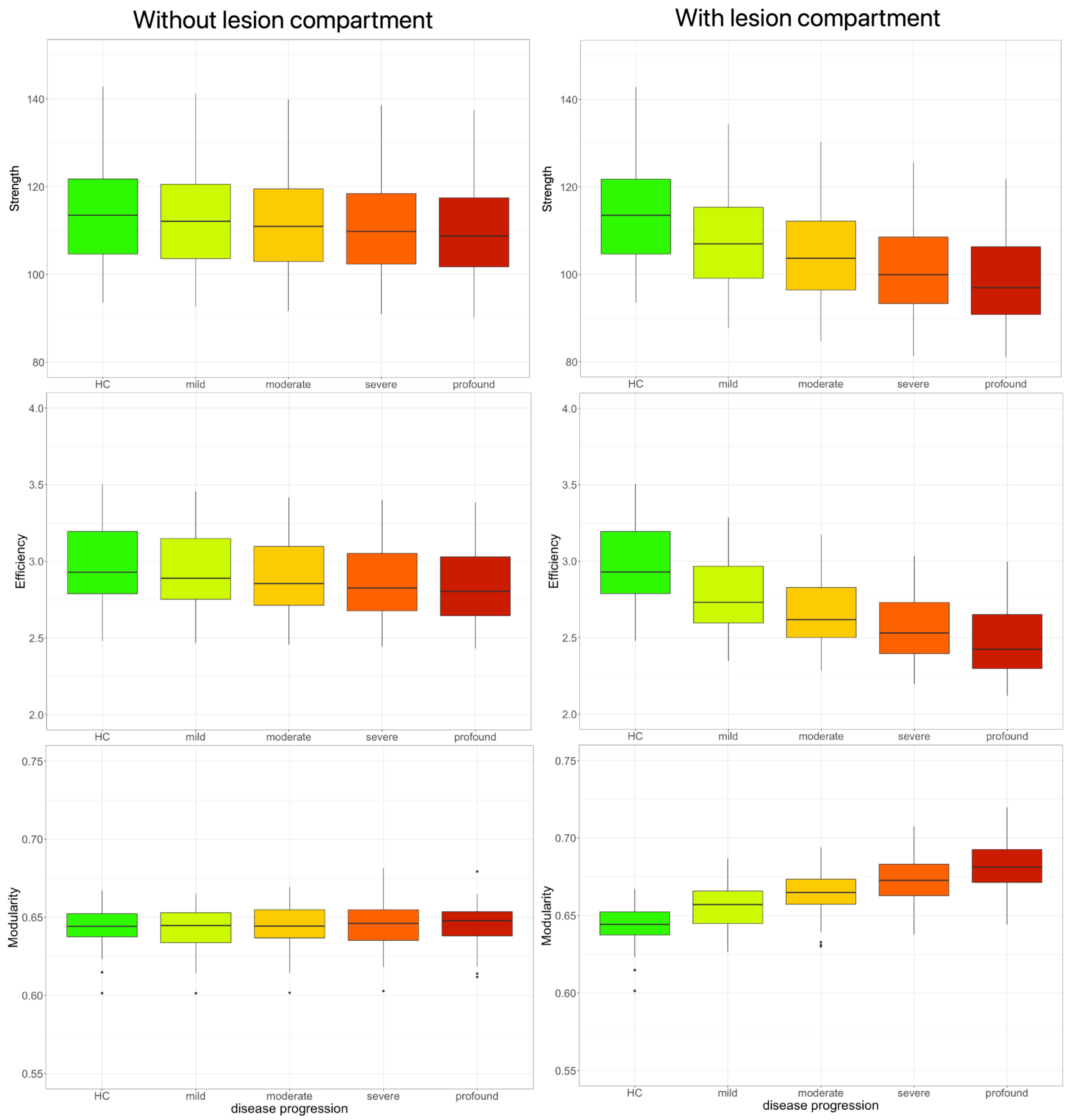

Figure 2. Comparison of global network metrics estimated with and without the lesion compartment on connectomes thresholded at 25%. The results of the permutation test are presented in Table 2.

	Group	Without lesion compartment<br>p-value	With lesion compartment<br>p-value
MEAN STRENGTH	mild	0.970	0.221
	moderate	0.999	0.404
	severe	0.312	**<0.001**
	profound	0.238	**<0.001**
GLOBAL EFFICIENCY	mild	0.972	**0.002**
	moderate	0.955	**<0.001**
	severe	0.331	**<0.001**
	profound	0.623	**<0.001**
MODULARITY	mild	0.982	**<0.001**
	moderate	0.999	**<0.001**
	severe	0.999	**<0.001**
	profound	0.999	**<0.001**

Table 2. The table present the results of the permutation test conducted on the connectomes thresholded by the 25%. The results shown in the table are visualized in the plots in Figure 2.

Experiments with different lesion volumes

The plot in Figure 3 shows the network metrics calculated from the connectome of the HCP data modeled by varying the lesion volumes (1%, 3%, 5%, and 7% with respect to the total WM).

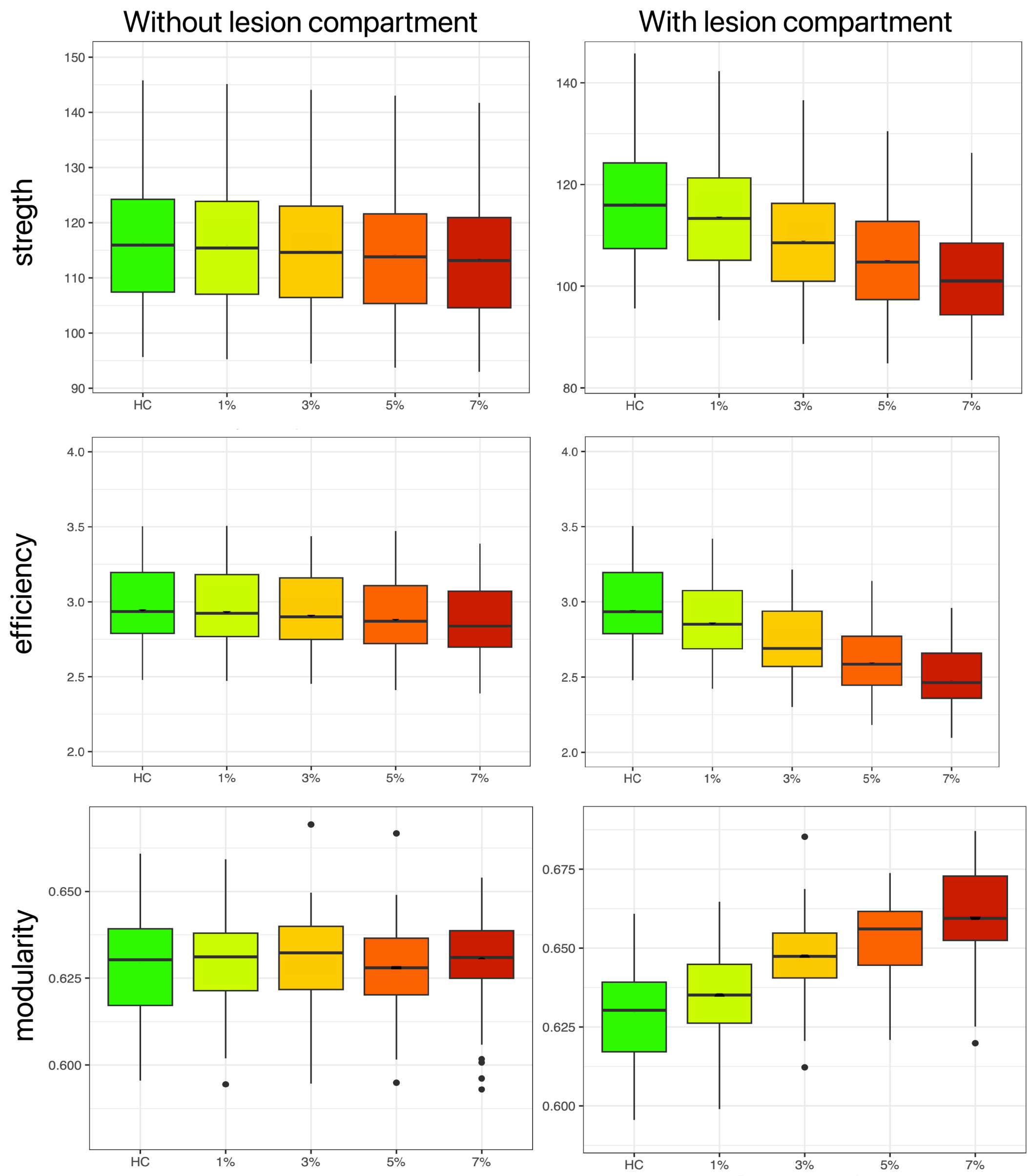

9 **Figure 3.** Comparison of global network metrics with and without the lesion compartment using HCP data modeled with different lesion volumes to assess
10 the model's sensitivity based on lesion charge rather than axonal density loss.

Example of lesion with different degree of axonal damage

The experiments shown in Figure 4 demonstrate that the method evaluates each voxel within the lesion mask independently. The left panel illustrates a concentric lesion with varying levels of axonal damage: 80% in the innermost square, 60% in the second concentric square, 40% in the third, and 20% in the outermost square. The second figure shows the lesion mask used for fitting the model, while the third figure displays the fitted contribution of the lesion compartment. It is evident that the contribution estimated by the model regarding the axonal damage is lower in the outer squares, such as those affected by edema, compared to the others. Specifically, the model's estimate of the lesion contribution increases in accordance with the extent of damage.

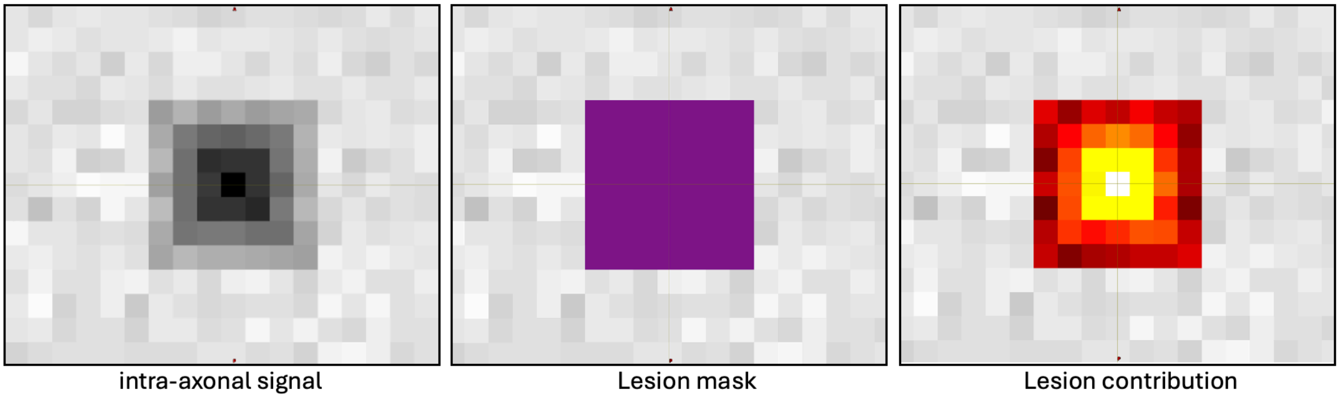

Figure 4. Example of the model application in case of non-uniform axonal damage. In the left is reported the simulated intra-axonal signal with different levels of axonal damage: 80% in the innermost square, 60% in the second concentric square, 40% in the third, and 20% in the outermost square. The second figure shows the lesion mask used for fitting the model, while the third figure displays the fitted contribution of the lesion compartment.
